# Electromagnetic Fields Exposure from Power Lines and Human Fertility

**Published:** 2019-05

**Authors:** Sedigheh ESMAILZADEH, Mouloud AGAJANI DELAVAR, Sayyed Asghar GHOLAMIAN, Amirmasoud AHMADI, Fatemeh HOSSEINPOUR HAYDARI, Mahbobe POURALI

**Affiliations:** 1. Infertility and Reproductive Health Research Center, Health Research Institute, Babol University of Medical Sciences, Babol, Iran; 2. Department of Electrical Engineering, Faculty of Electrical and Computer Engineering, Babol Noushirvani University of Technology, Babol, Iran; 3. Department of Biomedical Engineering, Faculty of Electrical Engineering, Iran University of Science and Technology (IUST), Tehran, Iran; 4. Department of Nursing, Babol University of Medical Sciences, Babol, Iran

## Dear Editor-in-Chief

Exposure to electromagnetic fields (EFM) is an indispensable part of our daily life and occurs in almost every situation. In developing countries, the main sources of these fields in the household are high-voltage power lines (1). In recent years, concern about its biological effects of people living next to high voltage power lines has been raised.

Some studies indicate adverse effects of EFM on the body system but the rule of EFM on reproduction system is unknown (2–4). In our previous study considering the effects of EFM on preterm labor was assessed. The adjusted OR for preterm birth among mothers living < 600 meters from high-voltage power line was 3.28 (95% Confidence Interval, CI, 1.37 to 7.85). In addition, a significant associations was observed between birth defect (adjusted OR = 5.05; 95% Confidence Interval, CI, = 1.52 to 16.78) and living < 600 meters from high voltage power line. No significant risk has shown for newborn death (5).

In our resent study raised a question if there is an impact on infertility from high-voltage power lines. For the evaluating the effects of EFM on infertility, a case control study was conducted. From 2012 to 2016, 462 subjects who had infertility problem referred to the Fatemezahra Infertility Clinic, Babol, Iran as case group and 471 subjects with no history of infertility, who were residents of the same city as control group were selected.

The ArcGIS software was used for calculation the nearest linear distance from the high-voltage power lines to the homes’ participants. Logistic regression analysis was used to assess the association between the distances to the high-voltage power line with infertility. Analyses were adjusted for residence, maternal age, maternal age at marriage, and the own occupation of men and women. A *P* ≤ 0.05 was considered to be significant. All confident intervals were calculated at the 95% level.

A significant associations were observed between infertility (adjusted OR = 3.52; 95% Confidence Interval, CI, = 2.32 to 5.33) and living less than 600 meters from a power line.

The results of two studies indicated that close residential proximity to high-voltage power line remained associated with preterm labor, birth defect, and infertility. The main limitation of these studies is no determined of the magnetic fields from sources other than high-voltage power lines, however the high-voltage power lines is a potential source of exposure. Therefore, additional work is needed to provide a better result in human fertility.
